# Immunoglobulin Replacement Therapy During COVID-19 Pandemic: Practical and Psychological Impact in Patients with Antibody Deficiency

**DOI:** 10.1007/s10875-023-01538-z

**Published:** 2023-06-26

**Authors:** Jesmeen Maimaris, Anjel O’Sullivan, Isabella Underhill, Ghiselle Green, Andrew Symes, David Lowe, Siobhan Burns, Mari Campbell, Reem Elfeky

**Affiliations:** 1 Institute of Immunity and Transplantation, University College London, London, UK; 2 Department of Immunology, Royal Free London NHS Foundation Trust, London, UK

**Keywords:** COVID-19, Immunoglobulin replacement, anxiety, depression, fatigue, antibody deficiency

## Abstract

**Purpose:**

The COVID-19 pandemic has impacted on how health services deliver care and the mental health of the population. Due to their clinical vulnerability, to reduce in-hospital attendances during the COVID-19 pandemic, modifications in immunoglobulin treatment regimens were made for patients with antibody deficiency. These patients were also likely to experience social isolation due to shielding measure that were advised. We aimed to investigate the impact of modifying immunoglobulin treatment regimen on infection and mental health burden during shielding restrictions.

**Method:**

Patients on immunoglobulin replacement therapy (IGRT) responded to a standardised questionnaire examining self-reported infection frequency, anxiety (GAD-7), depression (PHQ-8), fatigue (FACIT), and quality of life during the pandemic. Infection frequency and immunoglobulin trough levels were compared to pre-pandemic levels.

**Results:**

Patients who did not change treatment modality or those who received immunoglobulin replacement at home during the pandemic reported fewer infections. In patients who received less frequent hospital infusions, there was no significant increase in infections whilst immunoglobulin trough levels remained stable. There was no significant difference in anxiety, or depression scores between the treatment modality groups. Patients reported higher fatigue scores compared to the pre-COVID general population and in those discharged following hospitalisation for COVID.

**Conclusion:**

Changing immunoglobulin treatment regimen did not negatively impact infection rates or psychological wellbeing. However, psychological welfare should be prioritised for this group particularly given uncertainties around COVID-19 vaccination responsiveness and continued social isolation for many.

## Introduction

Patients with antibody deficiency are at greater risk of complications and chronic infection due to COVID-19 infection [1–3]. As a result, patients were encouraged to avoid non-essential journeys outside the house to reduce and mitigate the spread of COVID-19 infection — so called shielding guidance. Whilst many patients receiving immunoglobulin replacement therapy (IGRT) prefer home therapy due to the reduced impact on daily activities, some still prefer hospital-based treatment for reasons such as socialisation and perceived care [4, 5]. Prior to the pandemic, most of our patients receiving IGRT in hospital had an intravenous infusion every 3–4 weeks in hospital. In March 2020 at the outset of the pandemic, some patients opted instead to have weekly subcutaneous infusions, self-administered at home. The remainder on hospital IGRT received this every six weeks in hospital with the dose of immunoglobulin adjusted accordingly.

Research suggests that pandemics, such as COVID-19, are associated with a reduction in mental well-being across the population and reports in the UK general population from the first wave of the COVID-19 pandemic showed increased psychological morbidity [6–9]. This is likely linked to increases in socioeconomic inequalities, unemployment, physical distancing, and physical inactivity (Yu et al., 2020). Research investigating mental health in the UK during the COVID-19 pandemic is sparse and limited to the early stages and initial “lockdowns”. Niedzwiedz et al. (2020) describe an increase in psychological distress from 19.4% in April 2017 to 2019 to 30.6% in April 2020, as seen in the UK Household Longitudinal Study, and Kwong et al. (2020) report that the percentage of individuals with anxiety disorders almost doubled from 13% pre-pandemic, to 24% in April and May of 2020 [10, 11].

Pulvirenti et al. (2020) reported that patients with common variable immunodeficiency (CVID) reported higher anxiety and depression during the initial surge of COVID-19 in Italy [12]. However, there has been no research to date into the impact of COVID-19 on the mental health or quality of life of patients with primary and/or secondary antibody deficiency in the UK. COVID-19 has brought necessary changes in behaviour, especially for patients vulnerable to severe COVID-19, including a ‘forced’ change in treatment plans and reduced health services. It is unclear the effect that this has on their quality of life and/or mental health.

This study aimed to evaluate the clinical and psychological status of a large cohort of adult patients with antibody deficiency treated in one of the largest tertiary immunology centres in the UK during COVID-19 pandemic and assess the impact of changes in modality of receiving treatment on clinical and psychological wellbeing.

## Method

To measure the infection burden and psychological impact of shielding, all adult patients (> 18 years) with a diagnosis of antibody deficiency receiving IGRT at an immunology centre in London, UK, were approached via email to participate in an electronic survey in January 2021. Patients were asked for demographic details and to report the number of infections between March and December 2020 during which shielding guidance was in place. Respondents also completed standardised assessments of anxiety (Generalised Anxiety Disorder Assessment; GAD-7); depression (Patient Health Questionnaire; PHQ-8) and fatigue (Functional Assessment of Chronic Illness Therapy – Fatigue; FACIT-F) [6–8]. Finally, patients were asked to rate their perceived quality of life compared to pre-pandemic.

Based on IGRT treatment modality during the COVID pandemic in our centre, patients were divided into 3 main groups: patients with no change in therapy (no change), patients who switched to home therapy during the pandemic (home IGRT) and patients who opted to remain in hospital (hospital IGRT) but change the frequency of their treatment regimen (every 6 weeks instead of every 3 or 4 weeks). We also collected clinical data and mean trough IgG levels across the different groups of patients between March and December 2019, and March to December 2020. Indices of multiple deprivation (IMD) decile scores were collected from patient postcode records as a measure of socio-economic status, with 1 representing the most deprived and 10, the least.

SPSS v27 was used to analyse the data. Descriptive statistics were used to examine the psychological well-being of patients. An independent-sample *t*-test was used to compare anxiety (GAD-7), depression (PHQ-8) and fatigue (FACIT-Fatigue) of patients to healthy population norms during surge 1 of COVID-19. One-way independent ANOVAs were used to determine differences in psychological wellbeing (anxiety, depression and fatigue), number of infections, immunoglobulin IgG trough levels between patients on different treatment modalities: no change, home IGRT and hospital IGRT.

## Results

### Study Metrics

**Fig. 1:**
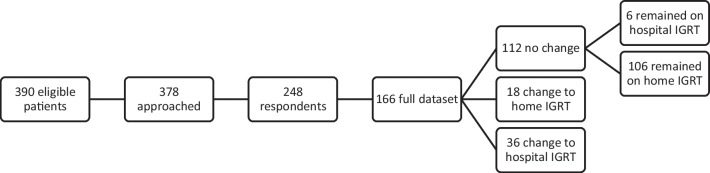
Recruitment and treatment pathway for participants

Three hundred seventy-eight of patients were approached, with 248 respondents. Incomplete or duplicate responses from patients were rejected. In the case of duplicate responses, the most recent response was accepted. Full data was available for 166 patients. 112 (67.5%) patients did not change their method of treatment (no change group); 6 of these patients continued to receive treatment in hospital under their pre-pandemic regimen and 106 remained on home immunoglobulin replacement regimens. Eighteen patients opted to switch to home IGRT (home IGRT) (10.8%) and 36 patients opted for hospital IGRT (hospital IGRT) (21.7%) but changed the frequency of their treatment regimen to receiving IGRT every 6 weeks instead of every 3 or weeks (Fig. 1).

### Patients’ Characteristics

Out of 166 patients in the study, 119 were receiving IGRT for primary antibody deficiency whilst 47 patients for secondary antibody deficiency. Each treatment group: no change, home IGRT, hospital IGRT received a comparable mean IGRT dose per week: 0.14, 0.14 and 0.13 g/kg/week respectively (Table 1).

**Table 1:** Patient demographics of 166 respondents to the electronic survey. Totals per group reported for sex and primary and secondary antibody deficiency with % in brackets. For age and IGRT dose median data shown with range in brackets. Totals reported in final column, with % of total in brackets; Age and IGRT dose reported as mean and standard deviation in brackets. IGRT, immunoglobulin replacement therapy. IMD, indices of multiple deprivation

Characteristic	No change (*n* = 112)	Home IGRT (*n* = 18)	Hospital IGRT (*n* = 36)	Total (*n* = 166)
Sex				
Female	47 (42%)	12 (67%)	17 (47%)	76 (45.8%)
Male	65 (58%)	6 (33%)	19 (53%)	90 (54.2%)
Antibody deficiency				
Primary	84 (75%)	12 (67%)	23 (64%)	119 (71.7%)
Secondary	28 (25%)	6 (33%)	13 (36%)	47 (28.3%)
Age (y)	52 (19–86)	57.5 (32–84)	58.5 (20–87)	52.5 (17.03)
IGRT dose (g/kg/week)	0.12 (0.01–0.44)	0.13 (0.07–0.29)	0.118 (0.06–0.57)	0.135 (0.07)
IMD decile score	7 (1–10)	8 (2–10)	7.5 (2–10)	6.6 (2.71)

### Clinical Outcomes

**Fig. 2:**
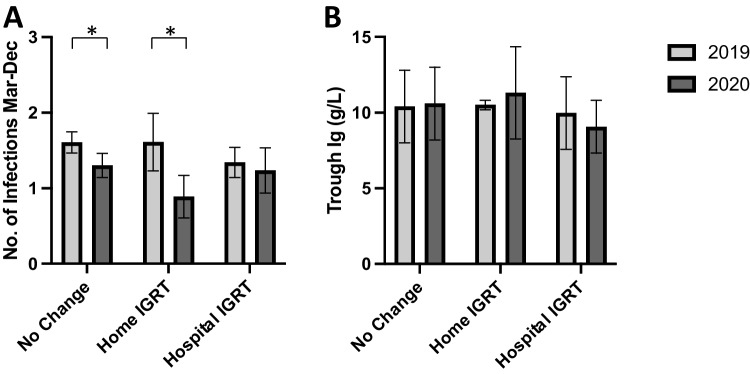
**A** Bar chart showing mean number of self-reported infections in treatment groups in 2019 compared to those in 2020. Error bars denote standard deviation, and * denotes *p*-value < 0.05. **B** Bar chart showing average immunoglobulin trough levels in treatment groups in 2019 compared to those in 2020. Error bars denote standard deviation

The number of reported infections between March and December 2020 was compared to the same time-period in 2019 in the 3 patient groups: no change, home IGRT, and hospital IGRT (Fig. 2). The mean number of infections decreased significantly between 2019 and 2020 in patients in the no change group ((1.61 ± 0.14) vs. (1.30 ± 0.16), *p* = 0.04) and patients in the home IGRT group ((1.61 ± 0.38) vs. (0.89 ± 0.28), *p* = 0.04). There was no change in number of infections among hospital IGRT group (mean 1.34 ± 0.20) in 2019 vs. (mean 1.24 ± 0.3) in 2020 (*p* = 0.09). Noticeably, immunoglobulin G trough levels remained stable among the hospital IGRT who moved to 6 weekly instead of 3–4 weekly IGRT (mean 9.974 g/L ± 2.39) in 2019 vs. (9.074 g/L ± 1.75) in 2020, *p* = 1.27.

### Psychological Wellbeing

#### Anxiety (GAD-7)

**Fig. 3:**
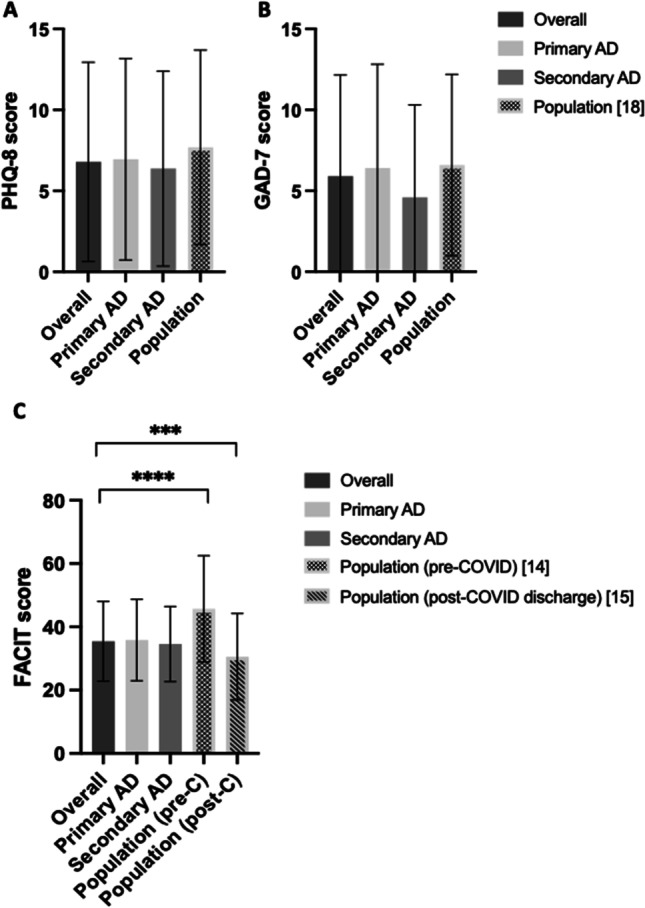
Patients’ psychological wellbeing scores. **A** PHQ-8 scores with mean with standard deviation depicted in overall study, patients with primary antibody deficiency (AD), patients with secondary antibody deficiency (AD), and population scores from Jia et al. [15]. **B** GAD-7 scores with mean with standard deviation depicted in overall study, patients with primary antibody deficiency (AD), patients with secondary antibody deficiency (AD), and population scores from Jia et al. [15]. **C** FACIT scores with mean with standard deviation depicted in overall study, patients with primary antibody deficiency (AD), patients with secondary antibody deficiency (AD), and population scores taken prior to COVID pandemic from Cella, et al. [14], and scores taken from a population recently discharged after COVID hospitalization, Harrison, et al. [16]. *** denotes *p*-value < 0.001 on multiple comparisons F-statistic (ANOVA)

Patients reported levels of anxiety similar to the general population (as rated during surge 1 of COVID-19 in the UK) (*t* (165) = -1.425, *p* = 0.16) [13]. 23.49% of patients had a level of anxiety that would meet Improving Access to Psychological Therapies (IAPT) criteria for a clinical diagnosis of anxiety (≥ 10) [14]. Whilst patients with primary antibody deficiency had higher anxiety scores than those with secondary antibody deficiency, this was not significant (*t* (164) = 1.78, *p* = 0.077) (Fig. 3).

#### Depression (PHQ-8)

Patients reported lower mood similar to the general population (as rated during surge 1 of COVID-19 in the UK) (*t* (165) = -1.887, *p* = 0.06) [13]. 26.51% of patients had a level of depression that would meet IAPT criteria for a clinical diagnosis of depression (≥ 10) [14]. There was no significant difference between PHQ-8 scores of patients with primary and secondary antibody deficiency (*t* (164) = 0.56, *p* = 0 0.47) (Fig. 3).

#### Fatigue (FACIT-Fatigue)

There were no data available for the general population on fatigue levels during the COVID-19 surges in the UK. However, patients reported significantly higher fatigue than the pre-COVID general population (*t* (165) = -10.51,* p* < 0.001) and significantly higher fatigue than patients who had recently been discharged from hospital following an admission for COVID (t (165) = 5.009, *p* < 0.001) [16, 17]. There was no significant difference between fatigue scores of primary and secondary antibody deficiency patients (*t* (165) = 0.64, *p* = 0.99) (Fig. 3).

#### Effect of Different Treatment Modality on Psychological Wellbeing

There was no significant difference in PHQ-8, GAD-7, and FACIT-Fatigue scores based on treatment category across groups 1–3, as seen respectively *F* (3, 162) = 1.16, *p* = 0.33, *F* (3, 162) = 0.74, *p* = 0.53, and *F* (3, 162) = 2.29, *p* = 0.08.

#### Quality of Life

Many participants (49.40%) stated that their quality of life had gotten worse or declined in comparison to before the COVID-19 pandemic.

## Discussion

This is the first study to provide data on the mental health of patients with antibody deficiency. It is also the first study in the UK examining the impact of the COVID pandemic on clinical and psychological wellbeing in patients with immunodeficiency. Our study demonstrated a reduction in infection frequency among patients with antibody deficiency during the COVID-19 pandemic, both in those who changed to home IGRT and in the group who had no change in IGRT (in which 94.6% received home IGRT). This is significant in our patients who are susceptible to recurrent infections and their associated sequelae. This effect has been noted in the general population more widely and is likely due to reduced circulating respiratory viruses [15, 18]. Interestingly, we noted stable trough levels in all groups including the hospital IGRT group who moved to a reduced frequency of IGRT administration. Our centre has continued to administer 6 weekly IGRT, given the benefits of reduced infections and fewer hospital visits. Further study, including health economic analysis, is required to determine the long-term effects of this change.

We also explored levels of anxiety, depression, and quality of life in adult patients with antibody deficiency during the COVID pandemic, and whether there was an impact of changes in modality of receiving treatment on psychological wellbeing. Our results showed similar, rates of anxiety and depression in January 2021 than the general population reported in the initial surges of COVID in March to May 2020 [13, 19–22]. On the contrary, rates of fatigue were higher in comparison to the pre-COVID population norm and interestingly even higher than the people who had been recently discharged from hospital for COVID [17]. These results for anxiety and depression are surprising, given previous research during non-COVID times has reported an increased prevalence of anxiety, depression and fatigue in patients with immunodeficiency [23]. Pulvirenti et al. (2020) noted raised levels of anxiety and depression in patients with immunodeficiency during the initial surge of COVID in Italy [12]. It is not yet known whether elevated levels of mental distress seen in the general population have continued as the pandemic has gone on, restrictions have been lifted and as people have been vaccinated. O’Connor and colleagues (2020) noted that levels of anxiety and depression decreased as time progressed within the pandemic, although their last point of data collection was May 2020 [20]. Participants completed our survey in January 2021, and it may be that the general population were reporting lower levels of anxiety and depression by this stage. It is worth mentioning that vaccination for COVID-19 in the UK started in December 2020. Unfortunately, we do not have comparison data for our cohort on psychological wellbeing pre-COVID. Regardless of this, it is important to note that nearly a third of patients (51/166, 30.1%) could have benefited from psychological support to manage anxiety and/or low mood.

No significant differences were found in levels of anxiety, depression, or fatigue between IGRT treatment modality. However, further investigation is needed to ensure patients are adequately supported in their choice of treatment modality going forward.

The results also suggest that just under a half of patients reported a decline in their quality of life since the start of the pandemic. Patients with immunodeficiency may have faced greater uncertainty and restrictions than the general population during the first and second surges, and the uncertainty associated with the pandemic remains high for this patient cohort as the efficacy of the vaccination is currently unknown. Elran-Barak and Mozeikov (2021) previously found that a third of immunocompromised patients reported feeling lonely before lockdown measures were even put in place, and that loneliness was a significant contributor to declining self-reported measures [24]. The increased shielding measures taken by this cohort of patients are likely to influence levels of loneliness and subjective mental/physical health. Further comparison research is needed, specifically longitudinal studies with a larger cohort that can closely map changes in healthcare and population health due to the pandemic. Poor mental health has been associated with poorer treatment adherence, less effective help seeking and higher rates of unhealthy behaviours, and these findings demonstrate the need for patients to have access to psychological support, and prompt psychological reassessment of patients with immunodeficiency [25, 26].

Our study has potential limitations. The study groups were not equal, with only a small group of patients moved to home IGRT. This is likely due to the logistical difficulty in arranging training sessions for home administration of IGRT at the onset of the pandemic. The study is a survey based retrospective study to understand psychological wellbeing in patients with antibody deficiency who were asked to shield during the COVID pandemic. The time frames of comparison between the patient cohorts (January 2021) and the general population were different (March to May 2020). However, the patient circumstances were likely to be similar during these times as patients were asked to limit social interactions due to perceived increased risk of COVID infection in immunodeficiency patients. Quality of life (QoL) was also collected to determine patient satisfaction criteria. This was not collated in a standardised mechanism, and thus was not included in statistical analysis and primary study outcomes. Pre-existing mental health conditions were not investigated in the survey. We used Indices of multiple deprivation (IMD) score as a weighted calculation of socio-economic status of our studied cohort.

Infections were self-reported by patients at the survey and at routine consultations with their physician. Infections were suspected when patients had symptoms such as increased cough frequency and sputum production, although individual symptoms vary between patients. Patients typically take a course of antibiotics upon suspected infection. No patient participants were hospitalised for COVID or other infections during the study period.

Whilst the reduction in reported infections in patients who have had or changed to home IGRT is encouraging, it should be balanced with shielding measures which are likely to influence levels of loneliness and subjective mental/physical health [19]. Further research is needed, specifically longitudinal studies with a larger cohort that can closely map changes in healthcare and population health. Our findings demonstrate the need for prioritised psychological welfare in patients with antibody deficiency, particularly given continued susceptibility of COVID-19 infection and reduced vaccination responsiveness.

## Data Availability

The datasets generated during and/or analysed during the current study are available from the corresponding author on reasonable request.

## Abbreviations

FACIT-F: Functional Assessment of Chronic Illness Therapy – Fatigue
GAD-7: Generalised Anxiety Disorder Assessment
IAPT: Improving Access to Psychological Therapies
IGRT: Immunoglobulin replacement therapy
IMD: Indices of multiple deprivation
PHQ-8: Patient Health Questionnaire

## References

[1] Meyts I (2021). Coronavirus disease 2019 in patients with inborn errors of immunity: an international study. J Allergy Clin Immunol.

[2] London J (2021). Severe COVID-19 in patients with B cell alymphocytosis and response to convalescent plasma therapy. J Clin Immunol.

[3] Brown LK (2022). Treatment of chronic or relapsing COVID-19 in immunodeficiency. J Allergy Clin Immunol.

[4] Espanol T (2014). Improving current immunoglobulin therapy for patients with primary immunodeficiency: quality of life and views on treatment. Patient Prefer Adherence.

[5] Jones GL (2018). What is the burden of immunoglobulin replacement therapy in adult patients with primary immunodeficiencies? A systematic review. Front Immunol.

[6] Löwe B (2008). Validation and standardization of the Generalized Anxiety Disorder Screener (GAD-7) in the general population. Med Care.

[7] Kroenke K (2009). The PHQ-8 as a measure of current depression in the general population. J Affect Disord.

[8] Yellen SB (1997). Measuring fatigue and other anemia-related symptoms with the Functional Assessment of Cancer Therapy (FACT) measurement system. J Pain Symptom Manage.

[9] Campion J (2020). Addressing the public mental health challenge of COVID-19. Lancet Psychiatry.

[10] Niedzwiedz CL (2021). Mental health and health behaviours before and during the initial phase of the COVID-19 lockdown: longitudinal analyses of the UK Household Longitudinal Study. J Epidemiol Community Health.

[11] Kwong ASF (2020). Mental health before and during the COVID-19 pandemic in two longitudinal UK population cohorts. Br J Psychiatry.

[12] Pulvirenti F (2020). Health-related quality of life in common variable immunodeficiency Italian patients switched to remote assistance during the COVID-19 pandemic. J Allergy Clin Immunol Pract.

[13] Jia R (2020). Mental health in the UK during the COVID-19 pandemic: cross-sectional analyses from a community cohort study. BMJ Open.

[14] National Collaborating Centre for Mental H. National institute for health and clinical excellence: Guidance, in generalised anxiety disorder in adults: management in primary, secondary and community care. Br Psychol Soc. 2011. https://pubmed.ncbi.nlm.nih.gov/22536620/. 22536620

[15] Olsen SJ (2021). Changes in Influenza and Other Respiratory Virus Activity During the COVID-19 Pandemic - United States, 2020–2021. MMWR Morb Mortal Wkly Rep.

[16] Cella D (2002). Fatigue in cancer patients compared with fatigue in the general United States population. Cancer..

[17] Harrison M et al. The interaction between fatigue and anxiety in people post hospitalisation with COVID-19. (0031–9406 (Print)).

[18] Reschen ME (2021). Impact of the COVID-19 pandemic on emergency department attendances and acute medical admissions. BMC Emerg Med.

[19] Pieh C (2021). Mental health during COVID-19 lockdown in the United Kingdom. Psychosom Med.

[20] O'Connor RC (2021). Mental health and well-being during the COVID-19 pandemic: longitudinal analyses of adults in the UK COVID-19 Mental Health & Wellbeing study. Br J Psychiatry.

[21] Shevlin M (2020). Anxiety, depression, traumatic stress and COVID-19-related anxiety in the UK general population during the COVID-19 pandemic. BJPsych Open.

[22] Hyland P (2020). Anxiety and depression in the Republic of Ireland during the COVID-19 pandemic. Acta Psychiatr Scand.

[23] Booker KBA, Haeney M, Bansal, Vieira A. Identifying risk for sub-optimal health-related quality of life and adjustment to illness in adults with primary antibody deficiency syndrome (PADS): Summary Report for the PiA 2007.

[24] Elran-Barak R, Mozeikov M (2020). One month into the reinforcement of social distancing due to the COVID-19 outbreak: subjective health, health behaviors, and loneliness among people with chronic medical conditions. Int J Environ Res Public Health.

[25] Sharpe M, Naylor C (2016). Integration of mental and physical health care: from aspiration to practice. Lancet Psychiatry.

[26] DiMatteo MR, Lepper HS, Croghan TW (2000). Depression is a risk factor for noncompliance with medical treatment: meta-analysis of the effects of anxiety and depression on patient adherence. Arch Intern Med.

